# Association of cardiometabolic risk factors and dental caries in a population-based sample of youths

**DOI:** 10.1186/1758-5996-2-22

**Published:** 2010-04-07

**Authors:** Roya Kelishadi, Shiva Mortazavi, Tavakol R Hossein, Parinaz Poursafa

**Affiliations:** 1 Paediatric Preventive Cardiology Department, Isfahan Cardiovascular Research Centre (WHO Collaborating Centre in Eastern Mediterranean Region), Isfahan University of Medical Sciences, Isfahan, Iran; 2 Paediatric Dentistry Department, Faculty of Dentistry, Torabinejad Dental Research Centre, Isfahan University of Medical Sciences, Isfahan, Iran; 3 Faculty of Dentistry, Torabinejad Dental Research Centre, Isfahan University of Medical Sciences, Isfahan, Iran; 4 Science and Research University, Tehran, Iran

## Abstract

**Background:**

Cardiovascular disease (CVD) risk factors begin from early life and track onto adulthood. Oral and dental diseases share some risk factors with CVD, therefore by finding a clear relation between dental diseases and cardiometabolic risk factors; we can then predict the potential risk of one based on the presence of the other. This study aimed to compare the prevalence of dental caries between two groups of age-matched adolescents with and without CVD risk factors.

**Methods:**

In this case-control study, the decayed, missing and filled surfaces (DMFS), based on the criteria of the World Health Organization, were compared in two groups of equal number (n = 61 in each group) of population-based sample of adolescents with and without CVD risk factors who were matched for sex and age group.

**Results:**

The study participants had a median age 13 y 5 mo, age range 11 y 7 mo to 16 y 1 mo, with male-to-female proportion of 49/51. We found significant difference between the mean values of DMFS, body mass index, waist and hip circumferences, as well as serum lipid profile in the case and control groups. Significant correlations were documented for DMFS with TC (r = 0.54, p = 0.02), LDL-C (r = 0.55, p = 0.01) and TG (r = 0.52, p = 0.04) in the case group; with LDL-C (r = 0.47, p = 0.03) in the whole study participants and with TC in control s(r = 0.45, p = 0.04).

**Conclusions:**

Given the significant associations between dental caries and CVD risk factors among adolescents, more attention should be paid to oral health, as one of the topics to be taken into account in primordial/primary prevention of cardiometabolic disorders.

## Introduction

Cardiovascular diseases (CVD) are the leading cause of mortality worldwide [1]. According to World Health Organization (WHO) estimates, by 2020, non-communicable diseases notably CVD will account for approximately three quarters of all deaths in the developing world [2]. There is a large body of evidence that CVD begin early in life, persist from childhood to adolescence and results in symptomatic diseases in adult life. Interest in childhood precursors to chronic diseases is increasing because the behavioural and biological risk factors for chronic diseases persist from childhood into adulthood [3,4].

New research is reinforcing the longstanding belief that an association exists between improper oral health and CVD [5-11].

There are known risk factors that make the cardiovascular system susceptible to diseases. Many of these risk factors are now brought to the life of young adolescents. Therefore, a high-risk life style from the early ages, leads to a higher risk of the heart diseases later in life. Diet, physical activity, and obesity have been highlighted among these risk factors [3,4]. There are also risk factors that lead to dental caries and poor oral health [12-14]. Dental caries is the most common chronic childhood disease. Even young children can be affected because a child's tooth is susceptible to decay as soon as it begins to erupt. Dental caries remains a significant problem in some populations, particularly certain racial and ethnic groups and in children from low social economic status regions of the world [14,15].

Various biological and socio-demographic factors, notably dietary factors, increase the risk of dental caries [16,17]. In turn, dental caries is suggested as one of oral health disorders related with health and well being in childhood [18] and CVD later in life as documented by a birth cohort study showing the associations of dental caries with angina pectoris [19].

Therefore finding association between dental caries and oral hygiene status with CVD risk factors in children and adolescents might be useful in preventive control measures against CVD from early life. The main objective of this investigation was to compare dental caries prevalence between two groups of matched adolescents with and without cardiometabolic risk factors.

## Methods

This study was conducted in the Paediatric Preventive Cardiology Department, Isfahan Cardiovascular Research Centre (ICRC), a WHO-Collaborating Centre in the Eastern Mediterranean region, and affiliated to Isfahan University of Medical Sciences, Isfahan, Iran. It was designed as a prospective case-control study to determine and compare decayed, missing, filled, surfaces (DMFS) in two groups of equal number (n = 61 in each group) of adolescents with and without CVD risk factors, considered as case and control groups, respectively. The two groups were matched for age group and sex distribution.

They were selected among participants of a community-based cross-sectional study of CVD risk factors in adolescents living in Isfahan, the second largest city in Iran. Those subjects with chronic disease and or long-term medication use or on specially recommended diets were excluded. The ICRC Ethics Committee (NIH Code: FWA 0000t8578) approved the study. Written informed consent was obtained from parents and oral assent from eligible adolescents.

The same team of paediatricians, general physicians and nurses examined all study participants. Based on the recommendations of Lohman et al. [20], three height (Ht) and weight (Wt) measurements were collected by calibrated instruments, and their average were used to compute the body mass index (BMI). BMI was converted to percentile by using the CDC (Centers for Disease Control and Prevention. Atlanta, GA 30333, USA) reference data [21]. Blood pressure (BP) was measured using mercury sphygmomanometers under standard protocol. The readings at the first and the fifth Korotkoff phase were taken as systolic and diastolic BP (SBP and DBP), respectively. The average of the two BP measurements was recorded and included in the analysis [22].

Participants were instructed to fast for 12 h before the screening. Compliance with fasting was determined by interview on the morning of examination while one of the parents accompanied his/her child. Blood samples were taken from the antecubital vein between 8:00 and 9:30 am. The blood samples were centrifuged for 10 min at 3000 rpm within 30 min of venipuncture. Fasting blood glucose (FBG), total cholesterol (TC), high density lipoprotein cholesterol (HDL-C), low density lipoprotein cholesterol (LDL-C), and triglycerides (TG) were measured by enzymatic method using autoanalyzer (Hitachi Model 902, Hitachi Ltd., Japan). HDL-C was determined after dextran sulphate-magnesium chloride precipitation of non-HDL-C [23]. Serum lipid profile and glucose were examined in ICRC central laboratory, which meets the standards of the National Reference laboratory (WHO Collaborating Centre in Tehran) and is also under an international quality control initiative.

CVD risk factors were defined according to the guidelines of the American Heart Association for identification of children and adolescents at high risk of CVD i.e. BMI ≥ 85th percentile for age and sex, TC >200 mg/dL, LDL-C>130 mg/dL, TG>150 mg/dL as well as systolic and diastolic blood pressure >90^th ^percentile for age, sex and height were considered to be elevated and HDL-C<35 mg/dL was considered to be low [24]. According to the recommendation of the American Diabetes Association, FBG ≥ 100 mg/dL was considered to be elevated [25]. Those adolescents with each of these risk factors were considered as the case group.

An expert dentist examined the decayed, missing, filled, surfaces (DMFS) of participants in both case and control groups blindly. Clinical examinations were carried out under indirect sunlight, using a sickle explorer, flat-surface mouth mirror after drying teeth with sterile gauze. Dental caries status was evaluated using the WHO caries diagnostic criteria for DMFS of teeth [26].

Data were transferred to SPSS statistical PC software version 11 (SPSS, Inc., Chicago, IL, USA) via a third person blind operator. The variables assessed were compared in the case and control groups by independent t and Mann-Whitney U tests. The correlation of DMFS with other variables was determined in the whole study participants and in the case and control groups separately by Spearman correlation test.

## Results

The study population comprised a total of 122 participants with median age 13 y 5 mo, age range 11 y 7 mo to 16 y 1 mo, and male-to-female proportion of 49/51. Table 1 presents the characteristics of participants of both groups. It shows significant differences between the mean values of DMFS, BMI, WC, HiC, TC, LDL-C, HDL-C, and TG between the case and control groups; whereas the mean FBG, DBP and SBP was not significantly different.

**Table 1:** Comparison of variables^a ^studied in the case and control groups

VARIABLES	CONTROL	CASE
Age (years)	13.5 ± 1.40	13.5 ± 1.40
Body mass index (kg/m^2^)*	20.7 ± 3.25	25.4 ± 9.72
Waist circumference (cm)*	77.8 ± 7.89	86.4 ± 13.13
Hip circumference (cm)*	91.6 ± 8.46	99.8 ± 12.16
Decayed, missing, filled, surface (DMFS)*	0.4 ± 0.03	0.8 ± 0.04
Diastolic blood pressure (mmHg)	64.3 ± 8.98	65.8 ± 9.33
Systolic blood pressure (mmHg)	103.3 ± 10.80	106.9 ± 10.62
Fasting blood glucose (mg/dL)	83.1 ± 5.36	84.9 ± 14.61
Total cholesterol (mg/dL)*	163.0 ± 21.16	194.1 ± 32.12
LDL- cholestrol (mg/dL)*	97.1 ± 19.33	121.4 ± 32.82
HDL- cholesterol (mg/dL)*	47.9 ± 9.40	43.6 ± 12.24
Triglycerides (mg/dL)*	99.6 ± 31.57	137.3 ± 65.11

^a^: mean ± SD; *, p < 0.05 between case and control groups

The Spearman correlation analysis showed significant relationships of DMFS with LDL-C (r = 0.47, p = 0.03) in the whole study participants and with TC in the control group (r = 0.45, p = 0.04). In the case group, significant correlations were documented for TC (r = 0.54, p = 0.02), LDL-C (r = 0.55, p = 0.01) and TG (r = 0.52, p = 0.04).

## Discussion

This study showed higher prevalence of dental caries among adolescents with higher risk of CVD and revealed significant associations between dental caries and CVD risk factors.

These results in the paediatric age group confirmed those of previous studies conducted with elder subjects that showed significant association of dental disease with CVD and its risk factors [27] Same results have been reported in a community-based study conducted among Swedish youths that showed higher number of caries free 15-year-old urban adolescents among those with low atherosclerosis risk [28].

A prevention program in Sweden studied dietary intake, CVD risk factors and dental caries in five cross-sectional groups of 15-year-old adolescents during 5 years. It revealed that dental caries were associated with increased BMI and CVD risk factors [29]. The association of dental caries with cardiovascular events remains controversial. While a study did not document any association between dental caries and acute ischemic stroke or transient ischemic attack [30], a birth cohort confirmed the associations of dental caries with angina pectoris [19].

Streptococcus mutans, the primary etiological agent of dental caries is also found as the most prevalent bacterial species in clinical samples of patients who underwent heart valve and atheromatous plaque surgery. Thus, the invasion of coronary artery endothelial cells by Streptococcus mutans OMZ175 underscores a potential role for these bacteria in the pathogenesis of CVD [31]. However, in addition to the bacterial aspects, dental caries is considered as a 'multifactorial' disease, such as many chronic diseases like cancer, CVD, and diabetes, which are the result of the interaction of many genetic, environmental and behavioural risk factors [32].

The correlation between BMI and dental caries scores has been frequently stressed [33-35]. This association might be caused by a carbohydrate rich diet, which rise both dental caries and CVD risks. This dietary pattern could be a factor affected by unhealthy lifestyle, notably poor diet. On the other aspect, body weight alone or included in multivariate statistic models has not been powerful predictor risk for dental caries prevalence and incidence in many studies [36-38]. Fluoride exposure and availability of oral health care services have been suggested as confounders for association between dental caries and childhood overweight [39].

Recent findings on the effect of inadequate glycemic control on increasing the incidence and progression of buccal alterations found in diabetic patients [40] underscores the importance of considering the association of oral health with chronic diseases. Our findings on correlation of DMFS with higher risk for CDVs can be suggested as another confirmatory evidence for the importance of considering multidisciplinary preventive measures against CVD from early life.

### Study strengths and limitations

Cross-sectional studies performed with convenience samples, such as the current one, cannot be generalized to the entire population. However, they can identify risk indicators of those parameters that are significantly associated with the condition being investigated. Whether these risk indicators would be confirmed to act as risk factor remains to be demonstrated in prospective studies. The main strengths of the study are its novelty in the paediatric age group, and its population-based design.

This study showed higher prevalence of dental caries among adolescents with higher risk of CVD and revealed significant associations between dental caries and CVD risk factors.

## Conclusions

This study showed significant associations between dental caries and CVD risk factors. Given that adolescents with high dental caries were at higher risk of these risk factors, more attention should be paid to oral health, as one of topics for prevention of cardiometabolic disorders. The most significant recommendation concluded from this study is to create a sense of responsibility among medical health professionals especially in the community of paediatricians, dentists, and general physicians to inform children and families about the short- and long-term hazards of having high DMFS and poor oral hygiene including their potential association with CVD risk factors. This concept should be taken into account in primordial/primary prevention of CVD.

## Conflict of interests

The authors declare that they have no competing interests.

## Authors' contributions

RK participated in the design of the study, provided the study participants, supervised the study, wrote and edited the manuscript, SM participated in the design of the study, supervised the study and edited the manuscript, TRH conducted the physical examination and participated in editing the manuscript, PP participated in writing and editing the manuscript. All authors read and approved the final manuscript.
